# In Vitro Testing of Alternative Synthetic and Natural Antiparasitic Compounds against the Monogenean *Sparicotyle chrysophrii*

**DOI:** 10.3390/pathogens10080980

**Published:** 2021-08-03

**Authors:** Ivona Mladineo, Željka Trumbić, Adrián Ormad-García, Oswaldo Palenzuela, Ariadna Sitjà-Bobadilla, Simona Manuguerra, Cristobal Espinosa Ruiz, Concetta Maria Messina

**Affiliations:** 1 Centre of Czech Academy of Science, Institute of Parasitology, Biology, 370 05 Ceske Budejovice, Czech Republic; 2 Department of Marine Studies, University of Split, 21000 Split, Croatia; ztrumbic@unist.hr; 3 Fish Pathology Group, Institute of Aquaculture Torre de la Sal, Consejo Superior de Investigaciones Científicas, 12595 Torre de la Sal, Spain; adrihighgrade@gmail.com (A.O.-G.); oswaldo.palenzuela@csic.es (O.P.); ariadna.sitja@csic.es (A.S.-B.); 4 Laboratorio di Biochimica Marina ed Ecotossicologia, Dipartimento di Scienze della Terra e del Mare DiSTeM, Università degli Studi di Palermo, Via G. Barlotta 4, 91100 Trapani, Italy; simona.manuguerra@unipa.it (S.M.); cespinosa31416@gmail.com (C.E.R.); concetta.messina@unipa.it (C.M.M.)

**Keywords:** anthelminthics, aquaculture, gilthead sea bream, monogenea, *Sparicotyle chrysophrii*

## Abstract

Gill monogenean *Sparicotyle chrysophrii* is considered the most detrimental fish parasite to the Mediterranean aquaculture. Treatment of sparicotylosis relies on frequent gill inspections correlated with the seasonal increase in seawater temperature, application of functional feeds, and treatments with formalin baths where permitted. While the latter is bound to be banned in Europe, other synthetic anthelminthics, such as praziquantel and ivermectin, are prone to induce resistance in the parasites. Therefore, we investigated, in vitro, 14 synthetic and natural compounds against adult *S. chrysophrii,* developing dose–response modelsm and estimated toxicity levels at 20%, 50%, and 80% parasite mortality. Bactericidal activity of target compounds was also tested in two important aquaculture bacteria; *Vibrio harveyi* and *V. anguillarum*, while their potential host toxicity was evaluated in gilthead seabream SAF-1 cell line. Synthetic compound bithionate sodium exerted the most potent toxicity against the monogenean, no host cytotoxicity, and a medium and high potency against two bacterial pathogens. In comparison, target natural compounds were approximately 20 (cedrol) or up to 154 times (camphor) less toxic for the monogenean. Rather than completely dismissing natural compounds, we suggest that their application in combination with synthetic drugs, especially if administered in the feed, might be useful in sparicotylosis treatment.

## 1. Introduction

Sparicotylosis is a detrimental parasitic disease affecting gilthead sea bream *Sparus aurata* that results in significant losses in Mediterranean-wide aquaculture. The causative agent, the polyopisthocotylean monogenean *Sparicotyle chrysophrii* Van Beneden et Hesse, 1863 (syn. *Microcotyle*), parasitises the gill epithelium, representing the only species within the genus *Sparicotyle* Mamaev, 1984. The clinical signs include lethargy, opercular hyperactivity, emaciation, inflammatory reddening with subsequent paling, and necrosis of gill filaments due to the severe damage of the gill epithelium, anaemia with low haemoglobin levels, and hypoxia [1,2]; the latter an assumed consequence of the monogenean haemophagus habit. The negative prognosis is usually conditioned by secondary bacterial and parasitic infections [3,4,5].

Registered prophylactic and therapeutic compounds against sparicotylosis that are safe, effective, environmentally friendly, and economically affordable are not available to date. Meanwhile, farmers have been combating the disease using baths containing formalin, hydrogen peroxide, freshwater, and eventually the illegal and hazardous permanganate treatment. A conducted questionnaire and consensual opinion of the Mediterranean farmers showed that a farmer applies on average 300 ppm formalin per 60 min baths to fish of 20–200 g, up to two times per week, synchronised with changing of the nets [6]. Typically, in a commercial outgrowth cycle of gilthead sea bream in sea cages, for producing 100,000 fish, 12 tons of formalin are used for controlling this disease. Apart of being banned in Italy, which likely is soon to be followed by the rest of EU, formalin application in sea cages has shown many drawbacks; a narrow therapeutic range, irritation of the gill epithelium, induction of hypoxia when applied in warm periods, toxicity to invertebrates in the water column, and sediment, toxicity, and carcinogenicity for the handling employees, as well as large operational costs [7,8], the latter, however, also being common for any other compound applied in sea cages.

Some widely known anti-parasitic treatments in aquaculture, such as distilled water, formalin, limoseptic^®^, hydrogen peroxide, chlorine, and praziquantel have been tested against *S. chrysophrii*, demonstrating that their efficacy varies between in vitro and in vivo treatments, as well as among the parasite’s life stages. Thus, the eggs showed to be the most resistant, followed by adults, while oncomiracidia were the most susceptible to the treatment [1].

In addition, an alternative strategy against sparicotylosis consists of feeds supplemented with iron to mitigate anaemia, or immunostimulants to boost the nonspecific host response, such as vitamin E, selenium, mannan oligosaccharides, ß-glucans, nucleotides, different plant extracts, fatty acids, and essential oils [9,10,11]. The best prophylactic strategy should therefore integrate functional feeding, routine gill inspection in seasons of expected parasite pressure (e.g., increased temperatures), net cleaning/changing and consequent treatment with an anti-parasitic compound.

Due to the limited knowledge about the efficiency of a broader spectrum of synthetic and natural compounds on *S. chrysophrii*, the aim of this study was to select a subset of already proven anti-parasitics and to test, in vitro, their effect on the adult monogeneans, as well as on a host cell line (gilthead sea bream *Sparus aurata* fibroblast; SAF-1). In addition, the compounds’ potential antimicrobial activity against *Vibrio harveyi* and *V. anguillarum*, two important pathogens in the Mediterranean aquaculture presenting comorbidity in sparicotylosis, was also evaluated.

## 2. Results

### 2.1. LC Assays and Dose–response Curves of Target Compounds

**Figure 1:**
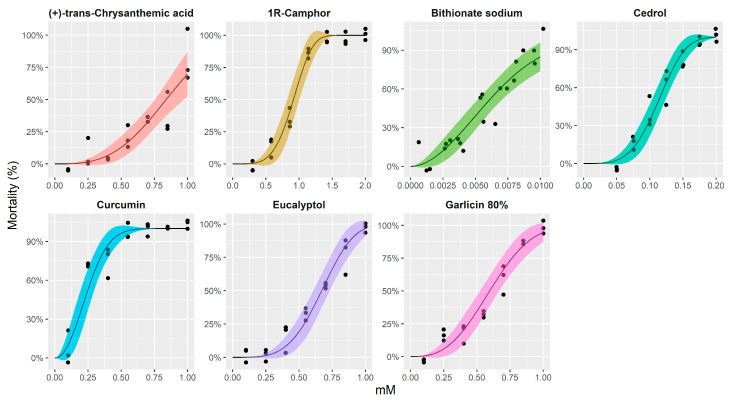
Dose–response curves with 95% confidence intervals for Sparicotyle chrysophrii adults treated with synthetic and natural compounds. The Weibull type 2 model for binomial response was fitted using the drc package for R.

The results of the dose–response models and estimated toxicity levels for some tested compounds, producing 20%, 50%, and 80% mortality with 95% confidence intervals for *Sparicotyle chrysophrii* adults, are shown in Figure 1 and Table 1. The remaining compounds were not considered for a scaled-down dose–response curve, as only the highest concentration (1 mM) showed 100% mortality at 24 h (bitoscanate, coumarine, diallyl sulfide, pyrethrins 50%) or the mortality was already reached after 1 h (*N*,*N*-diethyl-*M*-toluamide, 4-hexylresorcinol, monocrotaline). This would imply that their application in sea cages would request a prolonged fish exposure (>24 h), or very short treatment time (<1 h), respectively, posing technical impracticalities to the farmer.

In brief, natural compounds were observed to have lethal concentration (LC50) values 50% (LC50) higher (0.1–0.9 mM) than the only remaining synthetic compound, bithionate sodium (0.005–0.01 mM). Among the former, (+)-trans-chrysanthemic acid and camphor proved to be the least toxic, followed by eucalyptol and garlicin at 80%, while curcumin and cedrol proved to be the most toxic compounds for the parasite. The compound was designated as highly effective against adult monogenean if the LC50 reached in 4 h fit within the range of 0.1–0.01 mM concentration.

### 2.2. Viability of SAF-1 Cell Line after Exposure to Target Compounds

**Figure 2:**
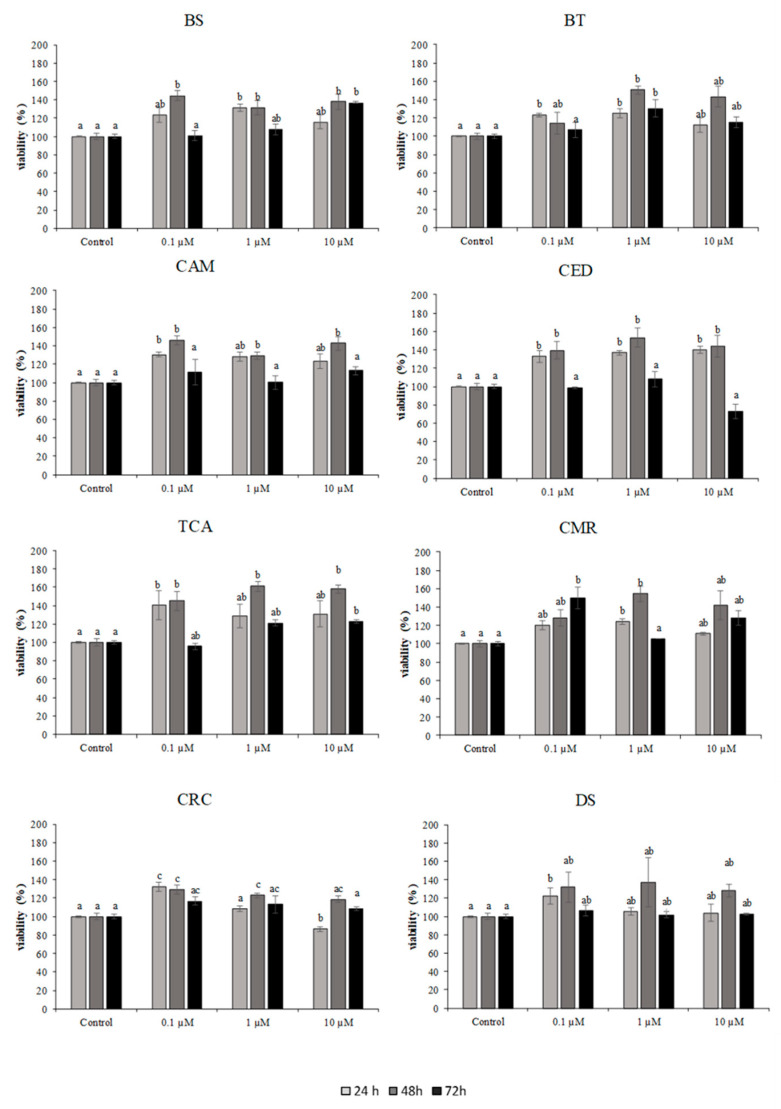
Cytotoxicity of SAF-1 cells exposed to different concentrations of natural and synthetic compounds (0.1–10 µM) for 24, 48, and 72 h. Bars represent the mean ± SEM (*n* = 6). Statistically significant differences (ANOVA; *p* < 0.05) were denoted using different superscripts letters (a, b, ab, c, ac). BS: bithionate sodium, BT: bitoscanate; CAM: camphor; CED: cedrol; TCA: (+)-trans-chrysanthemic acid; CMR: coumarin; CRC: curcumin; and DS: diallyl sulfide.

**Figure 3:**
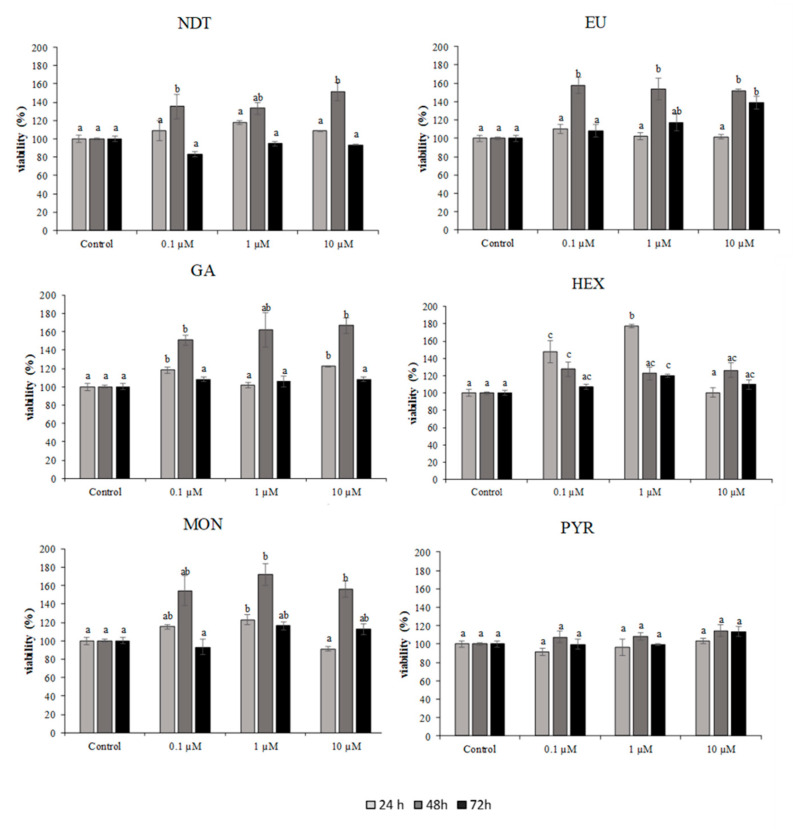
Cytotoxicity of SAF-1 cells exposed to different concentrations of natural and synthetic compounds (0.1–10 µM) for 24, 48, and 72 h. Bars represent the mean ± SEM (*n* = 6). Statistically significant differences (ANOVA; *p* < 0.05) were denoted using different superscripts letters (a, b, ab, c, ac). NDT: *N*,*N*-diethyl-*M*-toluamide; EU: eucalyptol; GA: garlicin 80%; HEX: 4-hexylresorcinol; MON: monocrotaline; and PYR: pyrethrins 50%.

Natural and synthetic compounds were tested in SAF-1 cells at concentrations between 0.1–10 µM. The results are shown in Figure 2 and Figure 3. Treatment with synthetic compounds; bithionate sodium, bitoscanate, *N*,*N*-diethyl-*M*-toluamide and 4-hexylresorcinol, in concentrations ranging 0.1–10 µM (24, 48, 72 h) did not induce a significant reduction in viability compared to control, highlighting the non-toxic effect of the compounds in this cellular system.

Among the natural compounds, only curcumin, at 10 µM for 24 h, exerted significant cytotoxicity.

### 2.3. Antimicrobial Assay of Target Compounds

Target compounds were also tested for their bactericidal activity against two important pathogens in fish aquaculture, *V. harveyi* and *V. anguillarum*.

In regard to *V. harveyi*, the synthetic compounds, bithionate sodium, *N*,*N*-diethyl-*M*-toluamide, and 4-hexylresorcinol showed a significant bactericidal activity at increased concentrations of the strain (Table 2). The natural compounds, such as eucalyptol, garlicin 80%, monocrotaline, and pyrethrins 50%, showed a significant antimicrobial activity at 10 µM (*p* < 0.05) (Table 3).

Regarding the bactericidal activity against *V. anguillarum*, the best antimicrobial activity was exerted by the synthetic compounds bithionate sodium, bitoscanate, and diallyl sulphide at 10 µM (*p* < 0.05). Among the natural compounds, the strongest antibacterial activity was demonstrated by cedrol, (+)-trans-chrysanthemic acid, coumarin, and curcumin (*p* < 0.05) (Table 2).

## 3. Discussion

Among the tested synthetic compounds, bithionate sodium [2,2′-sulfanediylbis(4,6-dichlorophenolate)] exerted the most potent toxicity (designated if compound’s LC50 reached in 4 h, fitted within the range of 0.1–0.01 mM concentration) against *S. chrysophrii* adults. Although not much information is available in respect to its anti-parasitic effect, its parent compound, bithionol, an aryl sulfide, is well-known for its fungicide [12] and anthelmintic properties, employed in agrichemistry and pharmacology. Bithionol was encompassed in an array of topical drugs against liver trematodes in humans [13], but it has been dismissed given its potent photosensitizing activity that results in serious skin disorders. Recently it has been tested as an anti-cancer drug based on its cytotoxic effects mediated through cell cycle arrest, reactive oxygen species (ROS) generation, and inhibition of autotaxin [14]. In aquaculture, bithionol and bithionol sulphoxide have been in vitro and/or in vivo tested against the monogeneans *Pseudactylogyrus* spp. [15], *Microcotyle sebastis* [16] and *Gyrodactylus* spp. [17], the flagellates *Ichthyobodo necator* [18,19], *Hexamita salmonis* [20] and *Neoparamoeba* spp. [21,22], and a ciliate, *Trichodina jadranica* [23], mostly showing desirable anti-parasitic effects, both as bath and oral treatments. Although bithionate sodium did not receive as much interest as bithionol, new findings suggest that it is able to inhibit cancer drug resistance targets [24] and cardiovascular diseases [25]. In addition, it has been shown as a very efficient potentiator of the novel eflux pump inhibitors in Gram-negative bacteria when combined with antibiotics, such as bactrin, streptomycin, ciprofloxacin, and doxycycline, helping to fight antibiotic-resistant infections [26]. In aquaculture such antimicrobial traits could be a useful synergistic effect combined with its anti-parasitic activity, for the mitigation of secondary bacterial infections frequently met during sparicotylosis [3,4,5]. Among the tested compounds, bithionate sodium showed a medium and high potency against *V. harveyi* and *V. anguillarum*, respectively, suggesting it as a candidate for further in vivo and in field testing.

Plant extracts and their highly diverse secondary metabolites represent a basis for traditional plant medicine in Africa, Asia, and America [27]. Their anti-parasitic mechanisms, usually complemented by anti-carcinogenic, anti-microbial, and cytotoxic mode of action are multiple. Some of them, such as berberine, sanguinarine, quinine, and furanoquinoline alkaloids, emetine, beta-carboline alkaloids, anthraquinones, furanocoumarins, and camptothecin interfere with the DNA replication and repair through intercalation, or impair DNA alkylation, such as aristolochic acid, cycasin, furanoquinoline alkaloids, furanocoumarins, pyrrolizidine alkaloids, and ptaquiloside. Other plant extracts disrupt cell membranes (mono- and sesquiterpenes, phenylpropanoids, isothiocyanates), affect microtubules integrity (colchicine, vinblastine, podophyllotoxin, sanguinarine, maytansine, rotenone, chalcones, and combretastatin), or neuronal signal transduction (many different alkaloids) [28]. In aquaculture, plant extracts have shown beneficial effects in respect to animal welfare [29], improvement of seafood shelf-life [30], or directly against pathogenic microorganisms, as in case of *Armoracia rusticana* natural compounds against *Vibrio anguillarum*, *V. harvey*, *V. alginolyticus*, *Aeromonas hydrophila*, *A. salmonicida*, *Photobacterium damselae* subsp. *piscicida*, *Tenacibaculum marinum*, and *Pseudomonas anguilliseptica* [31].

Expectedly, we observed that target natural plant extracts showed to be approximately 20 (cedrol) and up to 154 times (camphor) less toxic for the parasite (referred by LC50) than bithionate sodium. Therefore, exploring existing plant compounds to be used alone or in combination with synthetic drugs, might be useful in aquaculture, especially if administered in the feed.

Cedrol is a sesquiterpene alcohol encompassed in the essential oil of conifers (cedar oil), especially in the genera *Cupressus* (cypress) and *Juniperus* (juniper). It is widely used as a fragrance ingredient in decorative cosmetics, fine fragrances, shampoos, and toilet soaps, as well as in non-cosmetic products, such as household cleaners and detergents. Although it has been known for its anti-parasitic, nematicidal, and anti-fouling properties [32,33], it is also an oviposition attractant for gravid *Anopheles gambiae* and *A. arabiensis* mosquitos, a fact considered for “attract and kill strategy” in combating malaria [34]. It has not been previously tested against fish parasites, but has shown effectiveness against wild and drug-resistant *Leishmania donovani* amastigotes infecting humans at inhibitory concentration (IC50) of 1.5 μM. This is approximately 73 times lower than LC50 observed for *S. chrysophrii*, but cedrol still remains the most effective anti-parasitic natural compound tested herein. Interestingly, when incorporated and delivered within lipid nanocarrier, cedrol selectivity index (CC50/IC50; cytotoxic concentration 50) showed a 2.1-fold increase for wild-type *L. donovani* [33]. Being sparingly soluble in water (21.88 mg/L), which limits cedrol biodistribution and localization [35], the nanoparticle delivery system should be further explored as a potential mode of delivery through functional aquafeeds. However, this compound showed the highest reduction in sea bream fibroblasts’ viability at 10 µM concentration and 72 h of exposure, which needs to be considered in the course of a possible treatment, although such lengthy exposure is seldom considered in aquaculture. Interestingly, while the essential oil isolated from the heartwood of *Cunninghamia lanceolata* var. *konishii* (cedrol 58.3%) exhibited strong growth suppression against Gram-positive bacteria and yeast [36], the cedrol itself showed to be effective only against *V. anguillarum*.

Curcumin showed to be 2-fold less toxic for the monogenean compared to cedrol, a potent anti-*Vibrio anguillarum* agent (but ineffective against *V. harveyi*), and lacked negative effects on the sea bream cell line. This well-known polyphenolic compound of turmeric (*Curcuma longa* Linn.) has been previously tested in fish feed as an immunostimulator and booster in the antioxidant status and protein content [37,38]. Although it has been employed against many protozoan and few trematode infections in humans over the years [39], it has been recently introduced in aquaculture as an experimental and efficient treatment of the ciliates *Ichthyophthirius multifiliis* infecting carp [40] and *Philasterides dicentrarchi* infecting turbot [41].

Eucalyptol and garlicin 80% showed similar toxicity for *S. chrysophrii*, although approximately 3-fold lower than that of curcumin, and with the highest anti-*Vibrio harveyi* effect among tested natural compounds (ineffective against *V. anguillarum*). The monoterpene 1,8-cineole from eucalyptol inhibits acetylcholinesterase, mono-oxygenases, octopamine, or insect cytochrome P450, resulting in the compound’s anti-inflammatory and antioxidant properties exploited in aquaculture [42,43]. As an anti-parasitic, eucalyptol has been tested for treatment in poultry, bee, and cattle [44,45,46]. Observed eucalyptol anti-*Vibrio* and immunostimulative traits warrant its application through functional feed, possibly in combination with another more effective anti-parasitic as shown for the monogenean *Heterobranchus longifilis* [47]. Garlicin in contrast, has been tested against different gill parasites, being efficient against the monogenean *Anacanthorus penilabiatus* [48], the copepod *Lernanthropus kroyeri* [49], and the ciliates *Cryptocaryon irritans* [50], and *Ichthyophthirius multifiliis* [51]. However, it was inefficient against the monopisthocotylean monogenean *Neobenedenia* sp. [52] and *S. chrysophrii* [53], the latter corroborated by the current study.

Lastly, (+)-trans-chrysanthemic acid and camphor were the least toxic compounds for *S. chrysophrii*, e.g., approximately 1.2-fold less toxic than eucalyptol and garlicin 80%. Although the former produces esters belonging to class I pyrethrins [54], the effect was observed only against *V. anguillarum*. Similarly, camphor anti-parasitic properties have been proved in humans and domestic animals [55], but its inefficiency against *S. chrysophrii* does not make it a candidate for further research in respect to sparicotylosis.

In conclusion, bithionate sodium showed the best in vitro anti-parasitic and anti-*Vibrio* spp. properties, and no toxicity for the tested host cell line. The most potent natural plant extract cedrol was approximately 20-fold less toxic for the monogenean, moderately anti-bacterial, and moderately toxic to the host cell line during long-term exposure to very high concentrations. None of the tested natural plant extracts showed anti-bacterial properties against both aquaculture pathogens. Although preliminary, the results suggest that further testing of compounds in vivo through combined application in baths and functional feeds might be useful against sparicotylosis.

## 4. Materials and Methods

### 4.1. Fish and the Experimental Design

Gilthead sea bream (*Sparus aurata*) (donor fish = D; *n* = 200, average weight =100 g) naturally infected by *Sparicotyle chrysophrii* were captured using hand nets from production cages at a Mediterranean commercial site. They were placed in sea water containers supplemented with oxygen and transported to the facilities of the Institute of Aquaculture Torre de la Sal (IATS-CSIC), in Castellón (Spain). One half of the fish were placed into a 500 L tank connected to a RAS system, supplying additional 90 L-tanks holding naive, uninfected sea bream (recipient fish = R; *n* = 506, initial weight = 60 g). This enabled the transmission of the parasite to a larger number of fish reaching a moderate infection intensity, and avoiding heavy mortality associated to high parasite loads. The remaining D fish were kept in an open flow tank as a backup. Mean water temperature was 26.2 °C (never beyond 28 °C), and salinity was 37.5‰. More technical details on the procedure can be found in [56].

D and R fish were periodically checked for the presence of adult monogeneans, and if >5 worms were present on all gill arches from one side, fish were euthanised with an overdose of anaesthetic (MS-222, 1 g/10 L of water). The monogeneans were carefully collected from gill arches, placed in Petri-dishes with a thin layer of filtered sea water, and observed under the stereomicroscope, using fine needles.

Ethical statement: Fish manipulation and gills collection were carried out according to the Spanish (Royal Decree RD53/2013) and the current EU (2010/63/EU) legislations on the handling of experimental fish. All procedures were approved by the Ethics and Animal Welfare Committees of Institute of Aquaculture Torre de la Sal (IATS-CSIC), and “Generalitat Valenciana” (permit number 2018/VSC/PEA/0240).

### 4.2. LC Assays and Dose–response Curves of Target Compounds

The characteristics of all the tested compounds (all Sigma-Aldrich) are shown in Table 3.

Firstly, the adult monogeneans were exposed to a range of four concentrations of tested compounds encompassing the full dose–response range; each compound stock at 10 mM was serially diluted 10 (1 mM), 10^2^ (0.1 mM), 10^3^ (0.01 mM), and 10^4^ (1 µM) times. The target compounds have been historically shown to act at different orders of magnitude, and this enabled us to pinpoint the correct concentration range for each of them. Ten monogeneans per well (in triplicate) were added to 2 mL sterile seawater (SW) in 6-well plates, checked at the stereomicroscope for visible signs of mechanical damage, and exposed to selected concentrations in the dark at room temperature (Table 1). The number of live/dead parasites was recorded under the stereomicroscope at 1, 4, 12, and 24 h post-exposure. *S. chrysophrii* was considered to be alive if it performed any type of movement autonomously or 5 s after gentle probing with a dissection needle. Negative controls comprised the seawater with up to 0.5% of DMSO in total volume, the latter used as a solvent for each compound.

Subsequently, the monogeneans (*n* = 6, in triplicate) were exposed to a range of seven concentrations tailored according to each compound’s activity observed in preliminary tests. The range for each compound was scaled down to an appropriate order of magnitude so that each compound reached 100% mortality at 4 h post-exposure (Table 1).

For compounds that showed 100% mortality only at 24 h (bitoscanate, coumarine, diallyl sulfide, and pyrethrins 50%), or 100% mortality already after 1 h with the highest concentration (1 mM; *N*,*N*-diethyl-*M*-toluamide, 4-hexylresorcinol, and monocrotaline) no second scaled down LC assay was performed.

For the dose–response curve fitting, the drc package for R (v4.0.4) was used [57]. An appropriate nonlinear regression model was selected between log-logistic and Weibull type models, based on the Akaike information criterion (AIC) and lack-of-fit test criteria through the use of mselect function. The best scoring model was Weibull type 2 model (asymmetrical) with lower and upper limits fixed to 0 and 1, respectively, as mortality in all assays reached 100%. We compared a model distinguishing between different compounds, with an inferior one not incorporating this difference by the approximate F test, which showed that the dose–response curves were not identical (*p* < 0.0001). Model parameters and estimated lethal doses (20%, 50%, and 80%) were calculated with standard errors and 95% confidence intervals, based on the delta method, respectively, and adjusted for simultaneous inference through multcomp package [58]. Dose–response curves were visualised using ggplot2 library [59].

### 4.3. Viability of SAF-1 Cell Line after the Exposure to Target Compound

*Sparus aurata* fibroblast cell line SAF-1 (ECACC n°00122301) was cultured in 25 cm^2^ plastic flasks (Nunc, Darmstadt, Germany) in L-15 Leibowitz medium (Sigma, Haverhill, UK) supplemented with 10% fetal bovine serum (FBS), 2 mmol/L L-glutamine, 100 iu/mL penicillin, and 100 g/L streptomycin (all reagents from Sigma-Aldrich, Saint Louis, MO, USA).

Cells were grown at 25 °C under an 85% humidity atmosphere. Cells at 80% of confluence were detached with trypsin solution (0.25% of trypsin in PBS, pH 7.2–7.4) and pelleted by centrifugation (1000 rpm, 10 min, 25 °C). The cell suspension in complete medium was dispensed in a 96-multiwell plate at a density of 8000 cells/well and incubated for 24 h before the exposure to the compounds.

Cytotoxicity assay was performed in six replicates. Stock solutions were prepared by dissolving the compounds in dimethyl sulfoxide (DMSO), and then diluting in complete medium (final concentration of DMSO 0.1%) to obtain concentration of 0.1, 1, and 10 µM of target compounds.

Cells were then incubated for 24, 48, and 72 h in three different plates at 25 °C. Control samples received the same volume of culture medium and DMSO 0.1%, although this concentration of solvent is considered non-toxic [60,61].

The toxicity of the compounds was determined using the (3-(4,5-dimethylthiazol-2-yl)-2,5-diphenyltetrazolium bromide (MTT, Sigma-Aldrich) assay, according to [62], as reported in [61]. The results were expressed as viability percentage in respect to the controls (untreated cells).

The MTT assay is based on the reduction in the yellow soluble tetrazolium salt (3-(4,5-dimethylthiazol-2-yl)-2,5-diphenyltetrazolium bromide) (MTT, Sigma-Aldrich, Saint Louis, Missouri, USA) into a blue, insoluble formazan produced by the mitochondrial succinate dehydrogenase [63,64]. After incubation with the natural and synthetic compounds, SAF-1 cells were washed with phosphate buffer saline solution (PBS) and 200 mL/well of MTT (1 g/L) were added. After 4 h of incubation, cells were washed again, and the formazan crystals solubilized with 100 mL/well of DMSO. Plates were shaken (5 min, 100 rpm) in dark conditions and the optical densities (ODs) at 570 nm with background subtraction at 690 nm were determined in a microplate reader (Opsys MR™ Microplate Reader, USA). The percentage of viability was determined by formula:

Viability (%) = (OD of the tested sample/OD of the control sample) × 100

### 4.4. Bactericidal Activity of Target Compounds

Two bacterial fish pathogens, *Vibrio harveyi* and *V. anguillarum* were used to test the bactericidal activity of the target compounds, being previously isolated and identified as described in [65]. Lab strains were maintained at −20 °C in Tryptone Soy Broth (TSB, Difco Laboratories, Inc., Detroit, MI, USA) supplemented with 1% (*w*/*v*) NaCl (TSB1) and 50% (*v*/*v*) glycerol. Bacteria were cultured for 24 h at 25 °C in Tryptone Soy Agar (TSA, Difco Laboratories, Inc.) and Triptone Soy Broth (TSB, Sigma-Aldrich, St. Louis, MO, USA), both supplemented with 1.5% (*w*/*v*) NaCl. Bacteria in TSB medium were then cultured at the same temperature with continuous shaking (100 rpm) for 24 h. Exponentially grown bacteria were resuspended in sterile PBS and adjusted to the concentration of 10^8^ colony forming units (cfu)/mL. Each target compound (20 μL; concentrations 0.1, 1, and 10 µM) was added (in triplicates) to the wells of a flat-bottom 96-well plate, except for the positive control that consisted of 0.5% DMSO in PBS. Aliquots of 20 μL of the previously cultured bacteria were added, and the plate was incubated for 5 h at 25 °C. Then, 25 μL of MTT (1 g/L) was added to each well and the plate was additionally incubated for 10 min at 25 °C to allow the formation of formazan. The plate was then centrifuged (4500 rpm, 10 min) and the precipitate dissolved in 200 μL of DMSO. The solution (100 μL) from each well was transferred to another flat-bottom 96-well plate. The absorbance of the dissolved formazan was measured at 570 nm. Bactericidal activity was expressed as percentage of nonviable bacteria, calculated as the difference between absorbance of surviving bacteria compared to the absorbance of bacteria in positive controls (100%).

## Figures and Tables

**Table 1:** Dose–response model parameters (b = slope, c = lower limit, d = upper limit, and e = midpoint) with standard errors and toxicity levels for the parasite with 95% confidence intervals adjusted for simultaneous inference for *Sparicotyle chrysophrii* adults treated with synthetic and natural compounds.

Compound	Dose–Response Model Parameters		Toxicity Levels for Adult *S. chrysophrii* (mM)		
	Weibull Type 2, c = 0, d = 1				
	b (SE)	e (SE)	LC_20_ (95% CI)	LC_50_ (95% CI)	LC_80_ (95% CI)
Bithionate sodium	1.93 (0.36)	0.01 (0.001)	0.003 (0.002–0.005)	0.01 (0.005–0.01)	0.01 (0.01–0.01)
1R-Camphor	4.54 (0.91)	0.99 (0.05)	0.71 (0.53–0.89)	0.91 (0.78–1.05)	1.1 (0.94–1.26)
Cedrol	3.98 (0.66)	0.12 (0.01)	0.09 (0.06–0.11)	0.11 (0.1–0.13)	0.14 (0.12–0.16)
(+)-trans-Chrysanthemic acid	3.12 (0.69)	0.94 (0.07)	0.58 (0.43–0.74)	0.84 (0.69–0.99)	1.1 (0.79–1.41)
Curcumin	2.14 (0.44)	0.29 (0.03)	0.14 (0.06–0.23)	0.24 (0.16–0.32)	0.36 (0.26–0.45)
Eucalyptol	3.99 (0.7)	0.74 (0.03)	0.5 (0.38–0.63)	0.67 (0.58–0.76)	0.83 (0.71–0.94)
Garlicin 80%	2.79 (0.49)	0.68 (0.04)	0.4 (0.27–0.53)	0.6 (0.49–0.7)	0.81 (0.66–0.95)

**Table 2:** Bactericidal activity (%) of different natural compounds against *Vibrio harveyi* and *V. anguillarum*. *–statistically significant difference in antimicrobial activity at *p* < 0.05.

Co	*Vibrio Harvey*			*Vibrio anguillarum*		
	0.1 µM	1 µM	10 µM	0.1 µM	1 µM	10 µM
BTS	−8.8 ± 1.8	3.5 ± 11.6	40.0 ± 0.7 *	4.9 ± 11.8	49.2 ± 11.6	96.4 ± 1.7 *
BTN	−21.6 ± 6.0	−0.088 ± 16.3	13.1 ± 5.1	−10.8 ± 19.9	19.6 ± 3.7 *	30.5 ± 2.0*
CAM	−23.6 ± 2.6	7.7 ± 10.7	9.3 ± 7.4	−38.8 ± 22.0	−27.8 ± 19.0	−17.4 ± 9.3
CED	−44.9 ± 7.7	−23.8 ± 6.9	−2.4 ± 26.9	−6.9 ± 3.0	24.1 ± 8.0 *	30.8 ± 6.4 *
TCA	−47.7 ± 5.9	−25.4 ± 14.6	13.2 ± 6.5	−1.4 ± 13.0	8.15 ± 16.3	32.0 ± 9.0 *
CMR	−33.4 ± 3.9	−21.2 ± 13.3	−1.9 ± 7.6	1.5 ± 8.38	13.2 ± 13.4	17.3 ± 5.0 *
CRC	2.1 ± 15.0	7.6 ± 2.1	10.4 ± 4.5	9.5 ± 7.5	24.5 ± 14.6	34.9 ± 1.2 *
DAS	−36.6 ± 6.8	−36.4 ± 7.9	−1.6 ± 0.7	−11.3 ± 24.8	−5.8 ± 11.7	46.8 ± 4.0 *
NDT	−0.9 ± 8.7	4.5 ± 16.7	13.7 ± 0.6 *	−61.9 ± 29.0	−28.5 ± 24.1	9.1 ± 8.5
EUC	−2.7 ± 8.7	−1.9 ± 12.3	50.3 ± 6.8 *	−31.7 ± 155.9	−34.1 ± 10.5	23.6 ± 3.1
GAR	20.9 ± 19.9	28.7 ± 6.2 *	42.0 ± 1.3 *	−109.7 ± 84.2	−46.0 ± 17.2	−52.1 ± 31.4
4HR	19.6 ± 31.5	34.7 ± 10.1	67.5 ± 14.1 *	−121.1 ± 10.9	−76.1 ± 33.9	−55.5 ± 31.6
MCT	−4.9 ± 24.9	18.2 ± 10.9	40.1 ± 4.1 *	−73.0 ± 56.1	−46.9 ± 32.0	17.9 ± 0.6
PYR	−4.4 ± 21.0	5.6 ± 10.2	30.5 ± 5.2 *	−79.4 ± 14.2	−40.5 ± 11.7	−18.1 ± 4.2

Co: compounds; BTS: bithionate sodium; BTN: bitoscanate; CAM: camphor; CED: cedrol; TCA: (+)-trans-chrysanthemic acid; CMR: coumarin; CRC: curcumin; DAS: diallyl sulphide; NDT: *N*,*N*-diethyl-*M*-toluamide; EUC: eucalyptol; GAR: garlicin 80%; 4HR: 4-hexylresorcinol; MCT: monocrotaline; and PYR: pyrethrins 50%.

**Table 3:** Chemical characteristics of 14 tested compounds tested against monogenean *Sparicotyle chrysophrii* parasitising gills of the reared gilthead seabream (*Sparus aurata*), supplied at 10 mM concentration by Sigma-Aldrich.

Compound	M_r_	Solubility (mg) in 50 mL DMSO	Solubility (µL) in 50 mL DMSO	Origin	Scaled-Down Range (mM)
Bithionate sodium	356.05	178.03		Synthetic	0.001–0.01
Bitoscanate	192.26	96.13		Synthetic	1–5
Camphor (1R)	152.24	76.12		Natural	0.3–2
Cedrol	222.37	111.19	110.08	Natural	0.05–1
(+)-trans-Chrysanthemic acid	168.23	84.12	76.47	Natural	0.1–1
Coumarin	146.14	73.07		Synthetic	0.2–2.5
Curcumin	368.38	184.19		Natural	0.1–1
Diallyl sulphide	114.21	n/a	64.38	Synthetic	0.5–5
*N*,*N*-diethyl-*M*-toluamide	191.27	95.64	95.83	Synthetic	0.1–1
Eucalyptol	154.25	n/a	83.61	Natural	1–10
Garlicin 80%	146.27	n/a	90.7	Natural	0.1–1
4-Hexylresorcinol	194.27	97.14		Synthetic	0.05–1
Monocrotaline	325.36	162.68		Synthetic	0.1–1
Pyrethrins 50%	350	350	406.98	Natural	1–10

## Data Availability

Not applicable.
